# Case Report: The Experience of Managing a Moderate ARDS Caused by SARS-CoV-2 Omicron BA.2 Variant in Chongqing, China: Can We Do Better?

**DOI:** 10.3389/fmed.2022.921135

**Published:** 2022-06-09

**Authors:** Junnan Peng, Qiaoli Li, Jing Dong, Guodan Yuan, Daoxin Wang

**Affiliations:** ^1^Department of Respiratory and Critical Care Medicine, Second Affiliated Hospital of Chongqing Medical University, Chongqing, China; ^2^Department of Intensive Care Medicine, Chongqing Public Health Medical Center, Chongqing, China

**Keywords:** COVID-19, Omicron BA.2, ARDS, clinical characteristics, therapy

## Abstract

**Background:**

The severe coronavirus disease 2019 (COVID-19) pandemic is still raging worldwide, and the Omicron BA.2 variant has become the new circulating epidemic strain. However, our understanding of the Omicron BA.2 variant is still scarce. This report aims to present a case of a moderate acute respiratory distress syndrome (ARDS) caused by the severe acute respiratory syndrome coronavirus-2 (SARS-CoV-2) Omicron BA.2 variant and to discuss some management strategies that may benefit this type of case.

**Case Presentation:**

A 78-year-old man, who had four negative nucleic acid tests and a fifth positive, was admitted to our hospital. This patient was generally good upon admission and tested negative for anti-SARS-CoV-2 antibodies even after receiving two doses of the COVID-19 vaccine. On the 7th day of hospitalization, he developed a moderate ARDS. Improved inflammatory index and decreased oxygen index were primarily found in this patient, and a series of treatments, including anti-inflammation and oxygen therapies, were used. Then this patient’s condition improved soon and reached two negative results of nucleic acid tests on the 18th day of hospitalization.

**Conclusion:**

At-home COVID-19 rapid antigen test could be complementary to existing detection methods, and the third booster dose of COVID-19 vaccine may be advocated in the face of the omicron BA.2 variant. Anti-inflammatory and oxygen therapies are still essential treatments for ARDS patients infected with SARS-CoV-2 Omicron BA.2 variant.

## Introduction

Coronavirus disease 2019 (COVID-19) is caused by infection with severe acute respiratory syndrome coronavirus 2 (SARS-CoV-2) (1). The ongoing severe coronavirus disease 2019 (COVID-19) pandemic has affected over 200 countries and territories worldwide. During global circulation, SARS-CoV-2 has evolved many times to maximize its fitness in humans and environments. The World Health Organization (WHO) has designated five COVID-19 variants as Variants of Concern (VOCs): Alpha, Beta, Gamma, Delta, and the Omicron variants. Among VOCs, the Omicron variant is currently receiving great global attention, due to its incredible viral transmission and immunological escape (2). The Omicron variant has been divided into three sub-lineages, namely, BA.1 (B.1.1.529.1), BA.2 (B.1.1.529.2), and BA.3 (B.1.1.529.3) (3). On November 24th, 2021, the first Omicron variant (BA.1) was reported to the WHO by South Africa (4) and soon spread all around the world (5). Compared to Delta, this Omicron subvariant had 3.19 times effective instantaneous reproduction number (6) but lead to a lower risk of hospital admission and death (7). More recently, another Omicron subvariant (BA.2) was reported in Denmark, and Fonager et al. found significant differences in the mutation analysis of BA.1 and BA.2 (8). Subsequent studies revealed that BA.2 is substantially more transmissible and possesses a higher immune-evasive ability than BA.1 (9). Few patients infected with Omicron BA.2 progressed to severe cases, but some of them were transferred to ICU and died (10). To date, our knowledge of patients infected with the Omicron BA.2 variant, especially critically ill patients, is still scarce. This report aims to present the experience of managing a moderate acute respiratory distress syndrome (ARDS) caused by the SARS-CoV-2 Omicron BA.2 variant in Chongqing, China, and to discuss some management strategies that may have benefits for this type of case.

## Case Presentation

### Chief Complaints

On March 21st, 2022, the patient, a 78-year-old man, who had four negative nucleic acid tests and a fifth positive, was transferred to our hospital. He has been isolated in the designed hotel since March 17th, 2022, due to close contact with his daughter, a laboratory-confirmed COVID-19 patient. His daughter had worked with other laboratory-confirmed COVID-19 patients, who had close contact with the COVID-19 cases from other provinces.

### History of Present Illness

This patient described that he only had a mild cough and denied other symptoms, including fever, chills, sore throat, runny nose, chest pain, shortness of breath, muscle soreness, and fatigue. Chest computed tomography (CT) in the local hospital didn’t show typical signs of COVID-19, like glass opacity, bilateral multifocal ground, peripheral distribution, and multilobe involvement.

### History of Past Illness

This patient had a 20-year history of primary hypertension and paroxysmal atrial fibrillation. His daily medication included one 40 mg telmisartan, one 5 mg amlodipine, and one 2.5 mg warfarin, all taken orally. He had a history of tuberculous pleurisy, which had already been cured for many years (details unknown). He also had a history of gallstone surgery about 26 years ago (details unknown). He had received inactivated COVID-19 vaccine (Sinovac Coronavac) two times (August 19th, 2021 and September 9th, 2021).

### Personal and Family History

This patient and his wife did not have a significant personal history. They had one son and one daughter, and the family medical history was unremarkable for any significant disorders.

### Physical Examination and Laboratory Findings

Upon admission, the detected temperature, pulse, respiratory rate, blood pressure, and oxygen saturation of the patient were within normal range. The auscultation of the heart revealed an irregular heartbeat and the varied intensity of the first heart sound. At the same time, there were no apparent abnormalities observed in the examination of the lungs, abdomen, and limbs. The baseline laboratory findings of this patient showed no obvious abnormalities (Table 1). Moreover, he tested negative for both anti-SARS-CoV-2 immunoglobulin (Ig) G and IgM antibodies.

**TABLE 1:** Timeline of changes in laboratory parameters.

Days of hospitalization	1	2	3	4	5	6	7	8	9	10	11	12	13	14	15	16	17	18
WBC, 10^9^/L		4.82			4.73		6.56	11.32		11.08		5.43	4.63	5.87			8.87	8.94
Neutrophil, 10^9^/L		3.55			2.67		5.48	10.15		9.92		7.54	3.64	5.14			7.51	8.31
Neutrophil,%		73.7			56.4		83.5	89.7		89.5		72.0	78.7	87.7			84.7	92.9
Lymphocyte, 10^9^/L		0.72			1.45		0.81	0.86		0.48		1.12	0.64	0.36			0.93	0.48
Lymphocyte,%		14.9			30.7		12.4	7.6		4.4		20.6	13.8	6.1			10.5	5.3
Hemoglobin, g/L		125			129		117	121		102		115	105	108			102	101
Platelet, 10^9^/L		38.7			38.9		34.5	35.4		30.4		34.3	30.9	31.9			30.7	30.2
Hematocrit,%		93			88		69	80		148		195	210	223			220	223
CRP, mg/L		0.38				24.72	45.52	103.56		16.08		28.25		22.29			48.51	81.02
IL-6, pg/mL		5.63				103.14	1137	57.86		10		49.09		120.5			107.7	4.76
IL-10, pg/mL		4.03					12.42											
PCT, ng/mL		0.037				0.269	0.439	2.49		1.44		0.384		0.289			0.194	0.886
ALT, U/L		91			64		44	42		170		141		76			80	
AST, U/L		56			54		44	33		123		56		37			47	
TB, μmol/L		21.9			25.7		25.9	23.2		16.6		23.6		16.4			20.6	
DB, μmol/L		8.7			10.1		11	10.4		6.8		9.6		6.6			7.4	
LDH, U/L		239			238		279	351		303		334		307			373	
BUN, mmol/L		7.9					9.34	8.7		10.98		7.59		9.71			11.17	
Creatinine, μmol/L		81.9					99.1	98.3		87.2		89.2		78.6			87.8	
Total protein, g/L		72.4			71.6		64.9	72.5		62.6		66.7		59.9			65.2	
Albumin, g/L		45.7			45.1		39.2	37.6		31.5		35.7		33.4			35.9	
Prealbumin, mg/L		247			181		110	102		115		139		124			116	
APTT, s		33.7			39.9		56.1	70.3		34.4		31.6		21.4			50.1	
PT, s		14.2			12.8		19.0	30		17.7		13.1		11.2			32	
TT, s		19.5			19.5		91.5	36.9		20.7		16.8		18.5			20.8	
D-dimer, mg/L		0.29					0.18	0.19		0.16		0.57		4.85			0.44	
INR		1.22			1.1		1.63	2.57		1.52		1.13		0.97			1.94	
CK, U/L		98					221	320		134		101		39			35	
CK-MB, μg/L		7.0					12	20.7		12.2		12.3		8.9			19	
BNP, pg/mL							123											
Pro-BNP, pg/mL								1,546		2,276		3,545		1,451			2,487	
CD4 + lymphocyte, cells/μL		264			688					160		413		153				

*ALT, alanine transaminase; APTT, activated partial thromboplastin time; AST, aspartate aminotransferase; BNP, brain natriuretic peptide; BUN, blood urea nitrogen; CK, creatine kinase; CK-MB, creatine kinase isoenzyme MB; CRP, C-reactive protein; DB, direct bilirubin; IL, interleukin; INR, international normalized ratio; LDH, lactic dehydrogenase; PCT, procalcitonin; PT, prothrombin time; TB, total bilirubin; TT, thrombin time; WBC, white blood cell.*

### Genetic Testing

This patient had multiple positive reverse-transcription polymerase chain reaction (RT-PCR) results of SARS-CoV-2 nucleic acid during hospitalization [a cycle threshold (Ct) value <40 was defined as SARS-CoV-2 viral positive; Ct value of ORF1ab/N gene: March 21st, 10.22/7.46; March 22nd, 29.20/33.72; March 26th, 21.31/23.53; March 27th, 20.79/22.73]. The genome sequencing test confirmed the infection of the SARS-CoV-2 Omicron BA.2 variant.

### Primary Diagnosis

Based on the “Diagnosis and Treatment Protocol for Novel Coronavirus Pneumonia” (9th Trial Version) (11) and the discussion of our COVID-19 expert group, this patient was diagnosed with a confirmed case of COVID-19. Then this patient received traditional Chinses medicine for 15 days (starting on March 23rd) and appropriate treatment for primary hypertension and paroxysmal atrial fibrillation.

### Presence of Acute Respiratory Distress Syndrome

**FIGURE 1:**
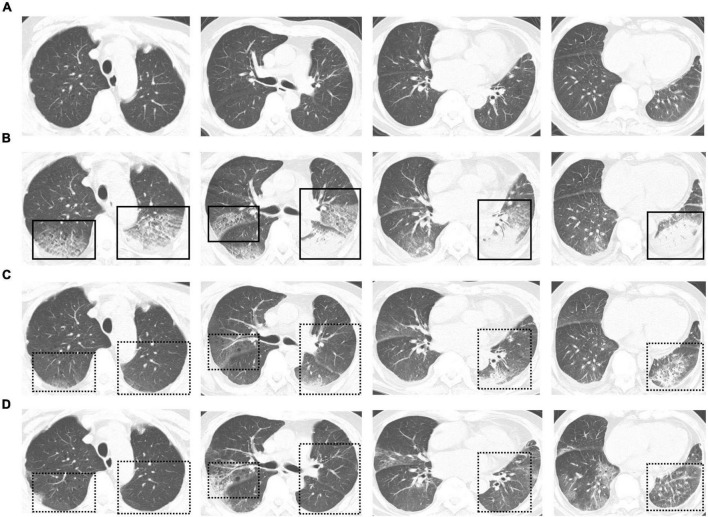
**(A)** Chest computed tomography (CT) scans obtained on March 25th. Few pulmonary ground-glass opacities can be seen. **(B)** Chest CT scans obtained on March 27th. Exudative infiltrates and consolidation, mainly on the right upper lung, left upper lung, and left lower lung, can be seen. An air bronchial sign can be seen in the consolidation shadow. **(C,D)** Follow-up chest CT scans obtained on March 30th **(C)** and April 3rd **(D)**. Pulmonary lesions were restored after treatment. Solid boxes show the new pulmonary lesions on chest CT. Dotted line boxes show restored pulmonary lesions on follow-up chest CT.

On March 25th, 2022, this patient developed a low-grade fever, with a maximum body temperature of 37.9°C. Chest CT revealed few pulmonary ground-glass opacities (Figure 1A). Later, on March 27th, this patient’s body temperature rose to 38.5°C, and he presented with shortness of breath. The oxygenation index greatly decreased, with a minimum number of 115 mmHg. Re-examination of chest CT showed the pulmonary lesions increased significantly (Figure 1B). According to the Berlin definition (12), this patient was diagnosed with moderate ARDS. Meanwhile, the interleukin (IL)-6 dramatically rose to 1,137 pg/mL, and other inflammatory parameters—including white blood cell, percentage of neutrophils and C-reactive protein—were all increased than at admission. The number of lymphocytes decreased gradually, with the lowest value of 0.36 × 10^9^/L. The coagulation parameters—including platelet, D-dimer, activated partial thromboplastin time, prothrombin time, and thrombin time—didn’t change a lot over time. And the laboratory parameters of heart, liver, and kidney function didn’t show signs of organ failure. Table 1 presents detailed results of laboratory parameters.

### Treatment

Considering this patient’s condition deteriorated rapidly, he was soon transferred into the intensive care unit. Our COVID-19 expert group conducted an urgent consultation, and a series of interventions were taken: (1) this patient had rapidly escalating oxygen requirements, therefore the high flow nasal cannula (HFNC) oxygen therapy (flow rate 50 L/min, oxygen concentration 50%) was administered. However, after several hours, his oxygenation index tended to decrease (oxygenation index = 115 mmHg), therefore we switched it to non-invasive ventilation (spontaneous/timed model, inspiratory positive airway pressure 10 cm H_2_O, expiratory positive airway pressure 6 cm H_2_O, fraction of inspired O_2_ 40%); (2) this patient had a persistent fever, increased inflammatory parameters and radiographically observed pulmonary lesions, therefore co-infected with bacterial should be considered. This patient received piperacillin-tazobactam intravenously (4.5 g thrice daily); (3) methylprednisolone (40 mg once daily) was used to suppress cytokine storm for 3 days; (4) thymalfasin (1.6 mg once daily) and immunoglobulin (20 g once daily) were used to modulate immunity for 3 days; and (5) anti-hypertension (adalat, 30 mg once daily), anticoagulation (edoxaban, 60 mg once daily), stabilizing ventricular rate (digoxin, 0.125 mg once daily), and nutritional supplementation were used and adjusted according to the actual situations.

### Short-Term Outcome

**FIGURE 2:**
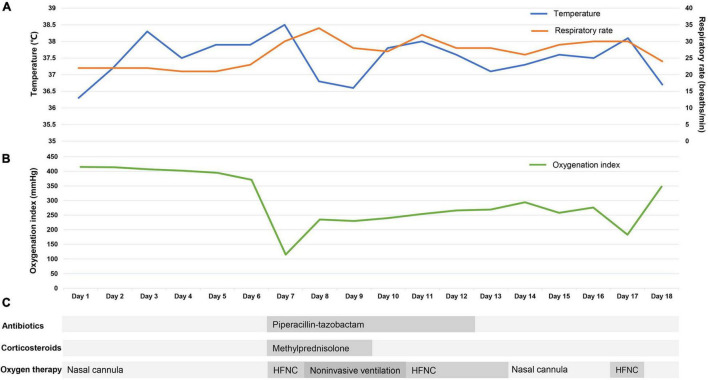
The timeline of essential clinical parameters and management of the patient. The X-axis represents the disease duration of the patient after hospital admission (days). **(A)** The Y-axis on the left represents the temperature value (blue line), and the Y-axis on the right represents the respiratory rate value (yellow line). **(B)** The Y-axis represents the oxygenation index value (green line). **(C)** The antibiotics, corticosteroids, and oxygen therapy of the patient. HFNC, high-flow nasal cannula oxygen therapy.

After treatment, this patient’s temperature fell to normal on March 28th, 2022. Although his temperature fluctuated in the following days, values were lower than 38.5°C. We observed a trend toward a reduced respiratory rate, and this patient had an improved symptom of shortness of breath. The oxygen index increased to 200–300 mmHg, and this patient was weaned from non-invasive ventilation on March 31st, 2022. Follow-up chest CT on March 30th and April 3rd showed the pulmonary lesions were further reduced (Figures 1C,D). Until April 3rd, this patient recovered well with no complications. Figure 2 presents the timeline of essential clinical parameters and management of this patient. On April 7th, this patient achieved two negative results of nucleic acid tests (Ct value of ORF1ab/N gene: April 6, 39.45/39.48; April 7, 35.36/39.68), and his oxygen index was 348 mmHg without fever and shortness of breath. Then, he was monitored for disease progress and continued the treatment program of the underlying diseases.

## Discussion

We describe here a case of a moderate ARDS caused by the SARS-CoV-2 Omicron BA.2 variant. This patient had four negative nucleic acid tests and a fifth positive, and his anti-SARS-CoV-2 antibodies were negative on admission. Although the initial symptoms of this patient were mild, his disease progressed rapidly and further developed to moderate ARDS. After timely and appropriate treatment, this patient’s prognosis was overall good. Although the COVID-19 pandemic has lasted for over 2 years, the management of the new SARS-CoV-2 variant, Omicron BA.2, remains a challenge for us (3). Thus, we intend to discuss some potential beneficial strategies from our experience and current literature in managing this type of case.

This patient should be considered a highly suspected case, due to close contact with his daughter, a confirmed COVID-19 patient. However, this patient tested negative four times for SARS-CoV-2 RNA until the fifth was positive. SARS-CoV-2 is infectious even in the incubation period (13), thus identifying cases of COVID-19 matters. Omicron replicated faster than all other SARS-CoV-2 variants in the bronchi but less efficiently in the lung parenchyma (14). This is compatible with the epidemiological observations that infection with Omicron produces higher infectivity but a less severe disease (7, 15, 16). No studies to date have reported any specific symptoms of infection caused by the Omicron BA.2 variant. But all variants are capable of causing severe symptoms (17), and some patients infected with Omicron BA.2 variant died (10). Proper and timely measures are always needed. Early detection of the infected cases, especially at the asymptomatic stage, can enable targeted interventions, thus reducing disease deterioration (18). Currently, detection of SARS-CoV-2 infection relies principally on two techniques: nucleic acid testing, which detects viral ribonucleic acid (RNA), and serological testing, which detects antibodies elicited against SARS-CoV-2 antigens. However, the high false-negative rate of RT-PCR was reported by several studies, and the anti-SARS-CoV-2 antibodies were increased at least 5 days after infection (19). Therefore, some experts have suggested repeated swab testing to diagnose COVID-19. More recently, a new approach, the at-home COVID-19 rapid antigen test, has been advocated for diagnosing COVID-19. Many countries have approved its use, and this approach showed reliable, user-acceptable, and safe abilities in some studies (20, 21). When people receive a reactive result, they can self-isolate, warn their contacts, and get timely treatment. It would be more cost-effective and time-saving to select COVID-19 positive patients if we combined this approach with nucleic acid testing and blood antibodies.

The vaccine is one of the most efficient tools to reduce rates of infections, hospitalizations, ICU admissions, and deaths for COVID-19 (22). In this case, this patient received two doses of inactivated COVID-19 vaccine before illness, but his anti-SARS-CoV-2 IgG and IgM antibodies were negative on admission, indicating no protective effect against COVID-19 disease. Vaccine effectiveness has been reported to wane within months of the second dose (23). The Food and Drug Administration (FDA) and the European Medical Agency (EMA) have authorized a third (booster) dose of COVID-19 vaccine to restore vaccine efficacy (24, 25). A large-scale study suggested that the three-dose SARS-CoV-2 mRNA vaccine is associated with lower rates of infection, hospitalization, and critical disease as compared to the two doses (26). Although Omicron is the most mutated SARS-CoV-2 variant to date, a booster vaccination program may provide satisfactory protection (27). Nemet et al. found a third COVID-19 booster vaccination efficiently neutralized infection with the omicron variant (geometric mean titer, 1.11 after the second dose vs. 107.6 after the third dose) (28). Another study from Hong Kong also supported that both SARS-CoV-2 mRNA and inactivated vaccine could protect against severe disease and death for Omicron BA.2, and the three doses offered higher protective ability and should be prioritized (29). Therefore, accelerating “mass and three-dose vaccination” is critical to control the COVID-19 pandemic and improve patient prognosis. Of note, studies predominantly supported the effectiveness of mRNA vaccines against the Omicron variant (27). Thus, evaluating the effect of other types of COVID-19 vaccines is still warranted.

During hospitalization, this patient progressively developed shortness of breath, and his oxygen index decreased to 115 mmHg. His initial chest image didn’t show lung involvement, and as the condition deteriorated, multiple pulmonary lesions, including ground-glass opacities and pulmonary consolidation, appeared in the lung. A previous study has found that the symptoms of SARS-CoV-2 were consistent with the changes in CT imaging (30). From our experience and the current literature (10, 31), the vast majority of patients infected with Omicron BA.2 were mild and few presented with pulmonary lesions. But once patients progress to severe disease, their pulmonary lesions may increase rapidly. Thus, chest CT remains an essential tool to assess the severity of Omicron BA.2. Laboratory examination showed the inflammatory index of this patient was significantly increased, and the IL-6 was up to 1,137 pg/mL on the day of ARDS diagnosis. Then the number of lymphocytes decreased, with the lowest value of 0.36 × 10^9^/L. This potent inflammatory response and associated immunosuppression lead to a cytokine storm, subsequently resulting in endothelial injury and hypercoagulability, ultimately leading to disease deterioration and even death (32). The time from admission to developing ARDS was 7 days for this patient, which was longer than wild-type SARS-CoV-2 (33). And this patient didn’t experience multiorgan failure, which was sometimes observed in critically ill COVID-19 patients (30, 33). Moreover, Gautret et al. found that patients infected with BA.2 had a higher mortality rate than BA.1 (10). But this may be due to the higher proportion of patients aged 65 years and more in the Omicron BA.2 group. As a comparison between ARDS induced by Omicron BA.2 and other SARS-CoV-2 variants is a topic of interest, more cohort studies are necessary to explore this issue. Corticosteroids are used to suppress the cytokine storm and ARDS for a variety of diseases including COVID-19 (34). In this case, this patient received intravenous methylprednisolone (40 mg, once daily) for 3 days from the day of developing ARDS, and his symptom and oxygen index improved soon. Meanwhile, corticosteroid treatment resulted in a rapid decrease in the IL-6, and IL-6 was closely associated with respiratory failure in COVID-19 (35). Although the dose and duration of corticosteroids in COVID-19 are still controversial (36), the early, low-dose (40 mg) and short-course (3–5 days) use could be feasible and effective from our experience. Moreover, this patient also received thymalfasin and immunoglobulin, which could block the inflammation and modulate the immune response (37). As excessive inflammation and immune suppression are considered to be the main mechanisms of COVID-19-induced ARDS (32, 34), these drugs have the potential abilities to improve the prognosis of these patients, especially older people.

Oxygen therapy and respiratory support are the essential treatments for COVID-19-induced ARDS and can improve patient outcomes (38). In this case, this patient received HFNC when his oxygenation levels decreased, and because his condition further worsened (oxygenation index = 115 mmHg), we switched it to non-invasive ventilation. After treatment, the oxygenation index of this patient rose to approximately 250 mmHg within 2 days. Our case supported that timely oxygen therapy is beneficial to improve oxygenation for patients with COVID-19-induced ARDS. We switched the oxygen therapy during the process of treatment, mainly due to the progressively decreased oxygen index and aggravation of breathlessness. We think the two indicators have the most value when considered to change respiratory support, which is consistent with the previous study (38). A recent study found that HFNC provided inconsistent oxygen index as to non-invasive or invasive ventilation (39), indicating great caution should be used during HFNC.

Our study has some limitations. First, this is a case report of a single patient. The inherent study limitations include lacking generalizability, inability to prove causality, and the risk of over interpretation of a single case. Second, we cannot entirely rule out mixed infection in this patient, even his blood antigen detection for chlamydia, mycoplasma, and influenza A and B virus was negative. However, as the patient’s condition improved considerably when the nucleic acid test turned negative, we are highly confident in the clinical diagnosis. Lastly, our study lacks a comparison between ARDS induced by Omicron BA.2 and other SARS-CoV-2 variants. This is because only one patient developed ARDS in our province, and we tried to provide detailed information to enable better a understanding of this case.

In summary, we have reported the experience of managing a moderate ARDS caused by the SARS-CoV-2 Omicron BA.2 variant. The take-home messages are (1) at-home COVID-19 rapid antigen test is complementary to existing detection methods, especially in the face of high infectivity of Omicron BA.2 variant, and (2) the three-dose (booster) COVID-19 vaccination, especially for the mRNA vaccines, may be needed when faced with the new challenge of Omicron BA.2 variant; and (3) anti-inflammatory and oxygen therapies are still essential treatment for ARDS patients infected with SARS-CoV-2 Omicron BA.2 variant. More research is needed to elucidate the biological differences between Omicron BA.2 and other SARS-CoV-2 variants and further guide clinical management.

## Data Availability Statement

The raw data supporting the conclusions of this article will be made available by the authors, without undue reservation.

## Author Contributions

JP, GY, and DW: conception and design. QL and JD: data curation. JP, QL, and JD: software and validation. All authors: writing—review and editing and final approval for publication.

## Conflict of Interest

The authors declare that the research was conducted in the absence of any commercial or financial relationships that could be construed as a potential conflict of interest.

## Publisher’s Note

All claims expressed in this article are solely those of the authors and do not necessarily represent those of their affiliated organizations, or those of the publisher, the editors and the reviewers. Any product that may be evaluated in this article, or claim that may be made by its manufacturer, is not guaranteed or endorsed by the publisher.

## References

[1] Lu R Zhao X Li J Niu P Yang B Wu H Genomic characterisation and epidemiology of 2019 novel coronavirus: implications for virus origins and receptor binding. *Lancet.* (2020) 395:565–74. 10.1016/s0140-6736(20)30251-8 32007145 PMC7159086

[2] Kuhlmann C Mayer CK Claassen M Maponga T Burgers WA Keeton R Breakthrough infections with SARS-CoV-2 omicron despite mRNA vaccine booster dose. *Lancet.* (2022) 399:625–6. 10.1016/s0140-6736(22)00090-3 35063123 PMC8765759

[3] Dhawan M Priyanka, Choudhary OP. Emergence of omicron sub-variant BA.2: is it a matter of concern amid the COVID-19 pandemic? *Int J Surg.* (2022) 99:106581. 10.1016/j.ijsu.2022.106581 35202859 PMC8860472

[4] Choudhary OP Dhawan M Priyanka. Omicron variant (B.1.1.529) of SARS-CoV-2: threat assessment and plan of action. *Int J Surg.* (2022) 97:106187. 10.1016/j.ijsu.2021.106187 34896627 PMC8656212

[5] Torjesen I. Covid-19: omicron may be more transmissible than other variants and partly resistant to existing vaccines, scientists fear. *BMJ.* (2021) 375:n2943. 10.1136/bmj.n2943 34845008

[6] Ito K Piantham C Nishiura H. Relative instantaneous reproduction number of omicron SARS-CoV-2 variant with respect to the Delta variant in Denmark. *J Med Virol.* (2022) 94:2265–8. 10.1002/jmv.27560 34967453 PMC9015237

[7] Nyberg T Ferguson NM Nash SG Webster HH Flaxman S Andrews N Comparative analysis of the risks of hospitalisation and death associated with SARS-CoV-2 omicron (B.1.1.529) and delta (B.1.617.2) variants in England: a cohort study. *Lancet.* (2022) 399:1303–12. 35305296 10.1016/S0140-6736(22)00462-7 PMC8926413

[8] Fonager J Bennedbæk M Bager P Wohlfahrt J Ellegaard KM Ingham AC Molecular epidemiology of the SARS-CoV-2 variant omicron BA.2 sub-lineage in Denmark, 29 november 2021 to 2 january 2022. *Euro Surveill.* (2022) 27:2200181. 10.2807/1560-7917.Es.2022.27.10.2200181 35272746 PMC8915403

[9] Mohapatra RK Kandi V Verma S Dhama K. Challenges of the omicron (B.1.1.529) variant and its lineages: a global perspective. *Chembiochem.* (2022) 23:e202200059. 10.1002/cbic.202200059 35322516 PMC9083815

[10] Gautret P Hoang VT Jimeno MT Lagier JC Rossi P Fournier PE The severity of the first 207 infections with the SARS-CoV-2 omicron BA.2 variant, in Marseille, France, december 2021-february 2022. *J Med Virol.* (2022). 10.1002/jmv.27760 [Epub ahead of print]. 35365865 PMC9088598

[11] National Health Commission. *Diagnosis and Treatment Protocol for Novel Coronavirus Pneumonia (Trial Version 9).* (2022). Available online at: http://www.gov.cn/zhengce/zhengceku/2022-03/15/content_5679257.htm (accessed March 16, 2022).

[12] Ranieri VM Rubenfeld GD Thompson BT Ferguson ND Caldwell E Fan E Acute respiratory distress syndrome: the Berlin definition. *JAMA.* (2012) 307:2526–33. 10.1001/jama.2012.5669 22797452

[13] Zou L Ruan F Huang M Liang L Huang H Hong Z SARS-CoV-2 viral load in upper respiratory specimens of infected patients. *N Engl J Med.* (2020) 382:1177–9. 10.1056/NEJMc2001737 32074444 PMC7121626

[14] Hui KPY Ho JCW Cheung MC Ng KC Ching RHH Lai KL SARS-CoV-2 omicron variant replication in human bronchus and lung ex vivo. *Nature.* (2022) 603:715–20. 10.1038/s41586-022-04479-6 35104836

[15] Wolter N Jassat W Walaza S Welch R Moultrie H Groome M Early assessment of the clinical severity of the SARS-CoV-2 omicron variant in South Africa: a data linkage study. *Lancet.* (2022) 399:437–46. 10.1016/s0140-6736(22)00017-4 35065011 PMC8769664

[16] Zhang J Chen N Zhao D Zhang J Hu Z Tao Z. Clinical characteristics of COVID-19 patients infected by the omicron variant of SARS-CoV-2. *Front Med.* (2022) 9:912367. 10.3389/fmed.2022.912367 35615088 PMC9125333

[17] Obireddy SR Guntakanti U Kowthalam A Marata Chinna Subbarao S Lai WF. Omicron: understanding the latest variant of SARS-CoV-2 and strategies for tackling the infection. *Chembiochem.* (2022):e202200126. 10.1002/cbic.202200126 [Epub ahead of print]. 35362644 PMC9083820

[18] Sun Q Qiu H Huang M Yang Y. Lower mortality of COVID-19 by early recognition and intervention: experience from Jiangsu Province. *Ann Intensive Care.* (2020) 10:33. 10.1186/s13613-020-00650-2 32189136 PMC7080931

[19] Arkhipova-Jenkins I Helfand M Armstrong C Gean E Anderson J Paynter RA Antibody response after SARS-CoV-2 infection and implications for immunity: a rapid living review. *Ann Intern Med.* (2021) 174:811–21. 10.7326/m20-7547 33721517 PMC8025942

[20] Møller IJB Utke AR Rysgaard UK Østergaard LJ Jespersen S. Diagnostic performance, user acceptability, and safety of unsupervised SARS-CoV-2 rapid antigen-detecting tests performed at home. *Int J Infect Dis.* (2022) 116:358–64. 10.1016/j.ijid.2022.01.019 35038598 PMC8759098

[21] Thomas C Shilton S Thomas C Batheja D Goel S Mone Iye C Values and preferences of the general population in Indonesia in relation to rapid COVID-19 antigen self-tests: a cross-sectional survey. *Trop Med Int Health.* (2022) 27:522–36. 10.1111/tmi.13748 35332616 PMC9115524

[22] Sikora D Rzymski P. COVID-19 vaccination and rates of infections, hospitalizations, ICU admissions, and deaths in the European economic area during autumn 2021 wave of SARS-CoV-2. *Vaccines.* (2022) 10:437. 10.3390/vaccines10030437 35335069 PMC8955952

[23] Levin EG Lustig Y Cohen C Fluss R Indenbaum V Amit S Waning immune humoral response to BNT162b2 Covid-19 vaccine over 6 months. *N Engl J Med.* (2021) 385:e84. 10.1056/NEJMoa2114583 34614326 PMC8522797

[24] U.S. Food and Drug Administration. *Coronavirus (COVID-19) Update: FDA Expands Eligibility for COVID-19 Vaccine Boosters.* Silver Spring, MD: U.S. Food and Drug Administration (2021).

[25] European Medicines Agency. *Comirnaty and Spikevax: EMA Recommendations on Extra Doses and Boosters.* (2021). Available online at: https://www.ema.europa.eu/en/news/comirnaty-spikevax-ema-recommendations-extra-doses-boosters (accessed March 16, 2022).

[26] Butt AA Talisa VB Yan P Shaikh OS Omer SB Mayr FB. Vaccine effectiveness of three vs. two doses of SARS-CoV-2 mRNA vaccines in a high risk national population. *Clin Infect Dis.* (2022):ciac178. 10.1093/cid/ciac178 [Epub ahead of print]. 35245940 PMC8903438

[27] Du Y Chen L Shi Y. Booster COVID-19 vaccination against the SARS-CoV-2 omicron variant: a systematic review. *Hum Vaccin Immunother.* (2022):1–19. 10.1080/21645515.2022.2062983 [Epub ahead of print]. 35499517 PMC9897643

[28] Nemet I Kliker L Lustig Y Zuckerman N Erster O Cohen C Third BNT162b2 vaccination neutralization of SARS-CoV-2 omicron infection. *N Engl J Med.* (2022) 386:492–4. 10.1056/NEJMc2119358 34965337 PMC8823651

[29] McMenamin ME Nealon J Lin Y Wong JY Cheung JK Lau EHY Vaccine effectiveness of two and three doses of BNT162b2 and CoronaVac against COVID-19 in Hong Kong. *medRxiv.* (2022) [Preprint]. 10.1101/2022.03.22.22272769 PMC9286709 35850128

[30] Shi H Han X Zheng C. Evolution of CT manifestations in a patient recovered from 2019 novel coronavirus (2019-nCoV) pneumonia in Wuhan, China. *Radiology.* (2020) 295:20. 10.1148/radiol.2020200269 32032497 PMC7233359

[31] Kim MK Lee B Choi YY Um J Lee KS Sung HK Clinical characteristics of 40 patients infected with the SARS-CoV-2 omicron variant in Korea. *J Korean Med Sci.* (2022) 37:e31. 10.3346/jkms.2022.37.e31 35040299 PMC8763884

[32] Zhou F Yu T Du R Fan G Liu Y Liu Z Clinical course and risk factors for mortality of adult inpatients with COVID-19 in Wuhan, China: a retrospective cohort study. *Lancet.* (2020) 395:1054–62. 10.1016/s0140-6736(20)30566-3 32171076 PMC7270627

[33] Wu C Chen X Cai Y Xia J Zhou X Xu S Risk Factors associated with acute respiratory distress syndrome and death in patients with coronavirus disease 2019 pneumonia in Wuhan, China. *JAMA Intern Med.* (2020) 180:934–43. 10.1001/jamainternmed.2020.0994 32167524 PMC7070509

[34] Quirch M Lee J Rehman S. Hazards of the cytokine storm and cytokine-targeted therapy in patients with COVID-19: review. *J Med Internet Res.* (2020) 22:e20193. 10.2196/20193 32707537 PMC7428145

[35] Jøntvedt Jørgensen M Holter JC Christensen EE Schjalm C Tonby K Pischke SE Increased interleukin-6 and macrophage chemoattractant protein-1 are associated with respiratory failure in COVID-19. *Sci Rep.* (2020) 10:21697. 10.1038/s41598-020-78710-7 33303843 PMC7729930

[36] van Paassen J Vos JS Hoekstra EM Neumann KMI Boot PC Arbous SM. Corticosteroid use in COVID-19 patients: a systematic review and meta-analysis on clinical outcomes. *Crit Care.* (2020) 24:696. 10.1186/s13054-020-03400-9 33317589 PMC7735177

[37] Wu M Ji JJ Zhong L Shao ZY Xie QF Liu ZY Thymosin α1 therapy in critically ill patients with COVID-19: a multicenter retrospective cohort study. *Int Immunopharmacol.* (2020) 88:106873. 10.1016/j.intimp.2020.106873 32795897 PMC7409727

[38] Shang Y Pan C Yang X Zhong M Shang X Wu Z Management of critically ill patients with COVID-19 in ICU: statement from front-line intensive care experts in Wuhan, China. *Ann Intensive Care.* (2020) 10:73. 10.1186/s13613-020-00689-1 32506258 PMC7275657

[39] Hultström M Hellkvist O Covaciu L Fredén F Frithiof R Lipcsey M Limitations of the ARDS criteria during high-flow oxygen or non-invasive ventilation: evidence from critically ill COVID-19 patients. *Crit Care.* (2022) 26:55. 10.1186/s13054-022-03933-1 35255949 PMC8899791

